# Starvation causes female-to-male sex reversal through lipid metabolism in the teleost fish, medaka (*Olyzias latipes*)

**DOI:** 10.1242/bio.050054

**Published:** 2020-04-03

**Authors:** Yuta Sakae, Akira Oikawa, Yuki Sugiura, Masatoshi Mita, Shuhei Nakamura, Toshiya Nishimura, Makoto Suematsu, Minoru Tanaka

**Affiliations:** 1 Division of Biological Science, Graduate School of Science, Nagoya University, Nagoya 464-8602, Japan; 2 Laboratory of Molecular Genetics for Reproduction, National Institute for Basic Biology, Okazaki 444-8787, Japan; 3 SOKENDAI (The Graduate University for Advanced Studies), Department of Basic Biology, Faculty of Life Science, Okazaki 444-8787, Japan; 4 RIKEN Center for Sustainable Resource Science, Metabolomics Research Group, Yokohama 230-0045, Japan; 5 Faculty of Agriculture, Yamagata University, Tsuruoka 997-8555, Japan; 6 Department of Biochemistry, Keio University School of Medicine, Tokyo 160-8582, Japan; 7 Department of Biochemistry, Showa University School of Medicine, Tokyo 142-8555, Japan; 8 Institute for Advanced Co-Creation Studies, Osaka University, Osaka 565-0871, Japan; 9 Department of Intracellular Membrane Dynamics, Graduate School of Frontier Biosciences, Osaka University, Osaka 565-0871, Japan; 10 Department of Genetics, Graduate School of Medicine, Osaka University, Osaka 565-0871, Japan

**Keywords:** Sex reversal, Metabolome, Pantothenate metabolism, Fatty acid synthesis

## Abstract

The teleost fish, medaka (*Oryzias latipes*), employs the XX/XY genetic sex determination system. We show here that the phenotypic sex of medaka is affected by changes in lipid metabolism. Medaka larvae subjected to 5 days of starvation underwent female-to-male sex reversal. Metabolomic and RT-qPCR analyses indicated that pantothenate metabolism was suppressed by starvation. Consistently, inhibiting the pantothenate metabolic pathway caused sex reversal. The final metabolite in this pathway is coenzyme A, an essential factor for lipogenesis. Inhibiting fatty acid synthesis, the first step of lipogenesis, also caused sex reversal. The expression of *dmrt1*, a critical gene for male development, was suppressed by starvation, and a *dmrt1* (Δ13) mutant did not show sex reversal under starvation. Collectively, these results indicate that fatty acid synthesis is involved in female-to-male sex reversal through ectopic expression of male gene *dmrt1* under starvation.

## Abstract (precis)

**Summary:** We investigated the effects of starvation on sex differentiation in medaka. Starvation caused female-to-male sex reversal through pantothenate metabolism, fatty acid synthesis and *dmrt1* expression.

## INTRODUCTION

Medaka (*Olyzias latipes*) is a small model organism that employs the XX/XY genetic sex determination system (Aida, 1921; Matsuda and Sakaizumi, 2016). XX medaka develop into females while XY medaka with *DMY*/*dmrt1bY* on the Y chromosome become males. In the wild, however, sex reversal (both male-to-female and female-to-male) is frequently observed among medaka (Shinomiya et al., 2004, 2010). It was recently reported that environmental factors may affect the phenotypic sex of medaka. High water temperature and cortisol treatment reportedly induced female-to-male sex reversal (Sato et al., 2005; Hattori et al., 2007; Hayashi et al., 2010; Kitano et al., 2012; Adolfi et al., 2019).

In medaka, several genes are reported to be sex related-genes. *foxl2* and *aromatase* display the female-specific expression in gonadal somatic cells from 0 and 5 dph, respectively (Nakamoto et al., 2006; Nakamura et al., 2009). Foxl2 is thought to have a function on the differentiation of granulosa cells, closely associated with the developing oocytes. Aromatase is the key enzyme for the conversion of testosterone to estrogen. The mutant of aromatase shows female-to-male sex reversal in medaka (Nakamoto et al., 2018). *gsdf* and *dmrt1* are encoded on the autosome but their expression are higher in and specific to male, respectively (Kobayashi et al., 2004; Shibata et al., 2010). These expressions are detected in gonadal somatic cells. The mutations to *gsdf* and *dmrt1* cause male-to-female sex reversal (Masuyama et al., 2012; Imai et al., 2015).

Fatty acid synthesis is the first step in *de novo* lipogenesis, and is a pathway for synthesizing saturated fatty acids ranging from butyric acid (4:0) to palmitic acid (16:0) by increasing the number of carbon chains in steps of two using malonyl Coenzyme A (CoA) and acetyl CoA as raw materials. This reaction is catalyzed by fatty acid synthase (FAS). FAS is a multi-functional enzyme that has seven domains and performs all the steps of fatty acid synthesis: initiation, extension, and termination. Briefly, first, carboxyl groups of acetyl-CoA and malonyl-CoA are transferred to the ACP domain. Next, the malonyl/acetyltransferase (MAT) domain is used for the initiation step. The ketoacyl synthase (KS), dehydrogenase (DH), enoyl reductase (ER), and ketoacyl reductase (KR) domains are all involved in the extension step. Finally, the thio-esterase (TE) domain releases the fatty acid (palmitic acid) during the termination step (Liu et al., 2010; Beld et al., 2015).

CoA is an essential metabolite for many metabolic pathways, including lipogenesis (Leonardi et al., 2005), and is synthesized through the pantothenate metabolism pathway (Jackowski and Rock, 1981; Leonardi et al., 2005). Pantothenate is known as vitamin B_5_ and animals are unable to synthesize it. This pathway is restricted at the reaction catalyzed by pantothenate kinase (Pank) (Jackowski and Rock, 1981; Robishaw et al., 1982). In vertebrates, four *Pank* genes are present, namely *Pank1*, *Pank2*, *Pank3* and *Pank4* (Leonardi et al., 2005; Zhou et al., 2001). In human, the expression patterns of the various Pank proteins differ from one to the next; Pank1 is expressed in the heart, liver and kidney; Pank2 is expressed ubiquitously; Pank3 is expressed in the heart, liver and muscle; and Pank4 expression appears to be ubiquitous (Zhou et al., 2001). *Pank4* was, however, reported to be a pseudogene due to the replacement of two important amino acid residues (Yao et al., 2019).

Many studies have reported that nutrition is intimately related to reproductive phenomena, including gonadal maturation, the reproductive cycle, gametogenesis and gamete fertility (Luquet and Watanabe, 1986; Robinson, 1996; Bindari et al., 2013; Fontana and Torre, 2016). For instance, many adult organisms show low reproductive activity under starvation conditions (Drummond-Barbosa and Spradling, 2001; Jaspers et al., 2014). Recently, starvation at the embryonic stage or at birth was found to reduce reproductive capacity in mouse (Wade and Schneider, 1992; Wang et al., 2017, 2018). However, little is known about the mechanism underlying the relationship between nutrition and sex determination and/or sex differentiation.

Here, we have found that starvation during sex differentiation caused female-to-male sex reversal in medaka. Two different metabolomic analyses and pharmacological treatments revealed that pantothenate metabolism and fatty acid synthesis are involved in sex reversal through the expression of the male-development gene *dmrt1*. Our results highlight an example of how the environment affects sex differentiation by triggering an internal metabolic change.

## RESULTS

### Starvation causes female-to-male sex reversal

We first determined the growth of medaka larvae under starvation conditions. The survival rate dramatically drops off 5 days after starvation (Fig. S1A). The difference in body length is also statistically apparent 3 days after the onset of starvation (Fig. S1B). The timing of this difference is consistent with the disappearance of the yolk ball (Fig. S1C).

**Fig. 1.**
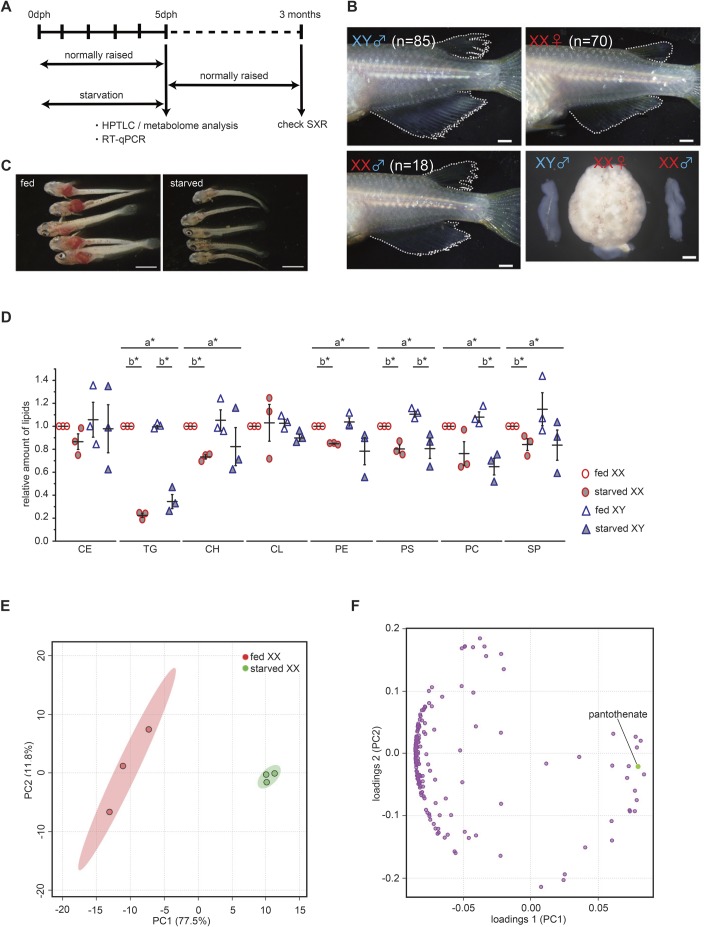
**5 days of starvation caused sex reversal and metabolic changes.** (A) Experimental design of starvation treatment. dph, day post hatching; SXR, sex reversal; HPTLC, high performance thin layer chromatography. (B) The appearance of dorsal and anal fin and gonad. Sex-reversed (XX male) individual indicates a male type of dorsal and anal fins with normal testis. The result of genotyping is shown in Table 1. Scale bars: 1 mm. (C) Oil Red O staining to visualize natural lipids in larva at 5 dph. Natural lipids (red signal) are invisible in 5 days-starved larvae. Scale bars: 1 mm. (D) The relative amount of lipids in 5 dph whole larvae (*n*=3). The total lipids were extracted from four larvae. The amount of TG is remarkably decreased by starvation. The values indicate the average and the bars indicate s.e.m. A two-way ANOVA followed by Dunnett’s test was used as statistical analysis. a*: *P*-value<0.05 in a two-way ANOVA. b*: *P*-value<0.05 in Dunnett’s test. CE, cholesterol esters; TG, triacylglycerols; CH, cholesterols; CL, cardiolipins; PE, phosphatidylethanolamines; PS, phosphatidylserines; PC, phosphatidylcholines; SP, sphingolipids. (E) Principal component analysis based on metabolome analysis (CE-TOF-MS, 175 metabolites). One dot indicates a data set of 20 larvae. Starved XX shows a clear difference from fed XX as seen in CE-TOF-MS analysis (fed *n=*3; starved *n=*3). Principal component, PC. (F) Loading plot of principal component analysis. A point indicates metabolite. Pantothenate shows a high contribution to the positive side of principal component 1. Principal component, PC.

Based on these results, we subjected larvae to 5 days of starvation after hatching and checked the sex phenotype of adults at 3–4 months of age (Fig. 1A). Interestingly, among medaka subjected to starvation, approximately 20% of genetic females (XX) showed a male type of dorsal and anal fins with normal testis (Fig. 1B, Table 1). Another medaka strain, d-rR, also showed female-to-male sex reversal under starvation conditions, although the ratio was relatively low (Fig. S1D; Table 1). We concluded that starvation during sex differentiation can cause female-to-male sex reversal in medaka.

**Table 1.**
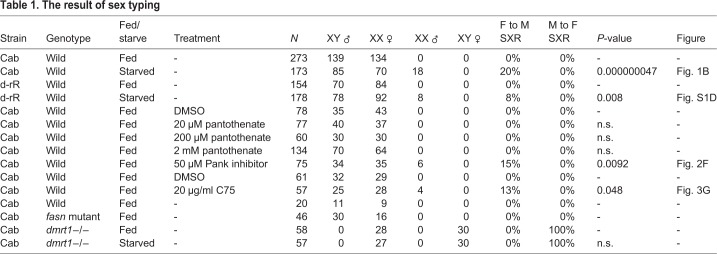
**The result of sex typing**

### Pantothenate metabolism is important for female sex differentiation

We expected that starvation would cause metabolic changes in medaka. To verify this, natural lipids were stained by Oil Red O (Fig. 1C). The stained natural lipid signals were dramatically decreased during 5 days of starvation. Consistent with this, high performance thin layer chromatography (HPTLC) analysis also indicated that triacylglycerol (TG) levels were markedly reduced in both sexes following starvation (Fig. 1D). Several other lipids were also reduced by starvation. This data indicates that the amount of lipid, especially TG, was affected by starvation.

**Fig. 2.**
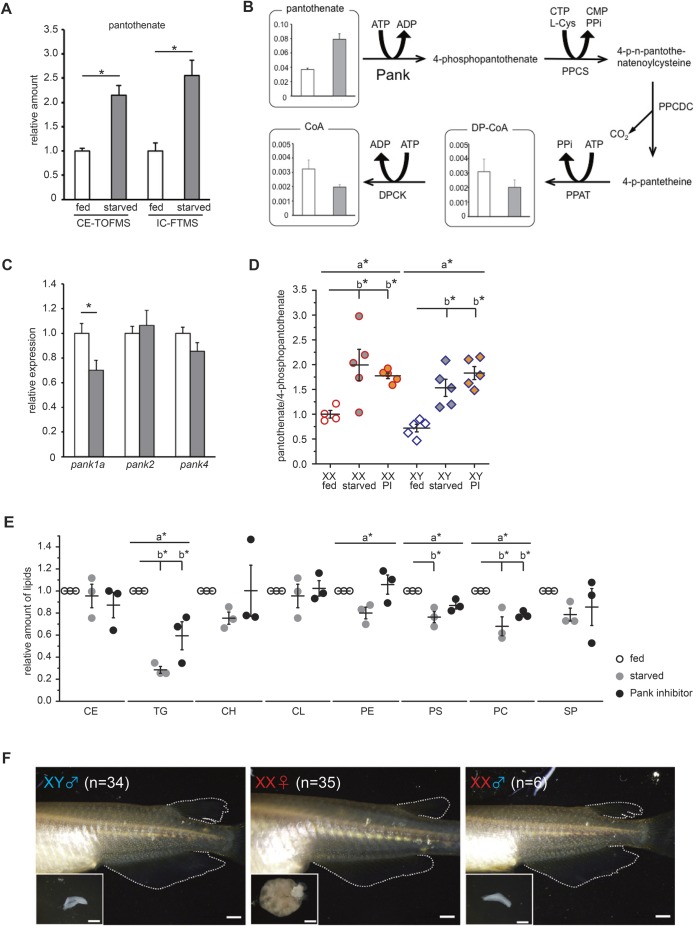
**Suppression of pantothenate pathway contributes to female-to-male sex reversal.** (A) The amount of pantothenate between normally feeding and starvation conditions. Whole XX larvae were analyzed based on CE-TOF-MS (an average of three experiments, each using 20 larvae) or IC-FTMS (an average of ten single larvae) at 5 dph. Pantothenate levels increase under starvation conditions. The values indicate the average and the bars indicate s.e.m. Student's *t*-test was used as statistical analysis. *P-value<0.05. (B) The amount of metabolites in the pantothenate pathway of whole XX larvae based on CE-TOF-MS analysis at 5 dph (an average of three experiments, each using 20 larvae). The vertical axis indicates the peak area of each metabolite in the CE-TOF-MS analysis. The values indicate the average and the bars indicate s.e.m. (C) *pank1a*, *pank2* and *pank4* transcript levels extracted from one whole XX larva at 5 dph (fed *n=*16, starved *n=*13) were determined by RT-qPCR. The expression level of *pank1a* is suppressed by starvation treatment. Transcript levels in starved samples were normalized to the average of those in fed samples for each gene. The values indicate the average and the bars indicate s.e.m. Student's *t*-test was used as statistical analysis. *P-value<0.03. (D) The ratio of pantothenate and 4-phosphopantothenate under several conditions. Larvae treated with Pank inhibitor accumulate pantothenate as seen under starvation conditions (XX fed *n=*4, XX starved *n=*5, XX Pank inhibitor *n=*5, XY fed *n=*5, XY starved *n=*5, XY Pank inhibitor *n=*5). The average pantothenate/4-phosphopantothenate ratio in fed XX was used to normalize each sample. PI, Pank inhibitor. The values indicate the average and the bars indicate s.e.m. A two-way ANOVA followed by Dunnett’s test was used as statistical analysis. a*: *P*-value<0.05 in a two-way ANOVA. b*: *P*-value<0.05 in Dunnett’s test. (E) The relative amount of lipids in 5 dph whole larvae (*n=*3). The total lipids were extracted from four larvae. The amount of TG is lowered by Pank inhibitor treatment, as seen under starvation conditions. The values indicate the average and the bars indicate s.e.m. A one-way ANOVA followed by Dunnett’s test was used used as statistical analysis. a*: *P*-value<0.05 in a two-way ANOVA. b*: *P*-value<0.05 in Dunnett’s test. See the legend of Fig. 1D for abbreviations. (F) Pank inhibitor treatment causes female-to-male sex reversal. Sex-reversed individual (XX male) indicates a male type of dorsal and anal fins with normal testis. The result of genotyping is shown in Table 1. Scale bars: 1 mm.

Next, we extracted water-soluble metabolites from 20 larvae (5 days post hatching, dph) that were either feeding normally or raised under starvation conditions. The extracted metabolites were applied to CE-TOF-MS. A total of 175 metabolites were identified, and principle component analysis (PCA) of the larvae indicated that two clusters, fed larvae and starved larvae, are clearly separated on an X (principle component 1) axis (Fig. 1E). Metabolites from single larvae (5 dph) were also analyzed by IC-FTMS (anionic metabolites) and LC-MS/MS (cationic metabolites). This analysis again resulted in two different groups along an X (principle component 1) axis: normally fed larvae and starved larvae (Fig. S1E). Pantothenate was the common metabolite according to the value of loading among the metabolites (Fig. 1F; Fig. S1F; Table 2). Intriguingly, CE-TOF-MS and IC-FTMS analysis showed that starved XX had higher pantothenate levels than fed XX despite medaka could not synthesize the pantothenate (Fig. 2A).

**Table 2.**
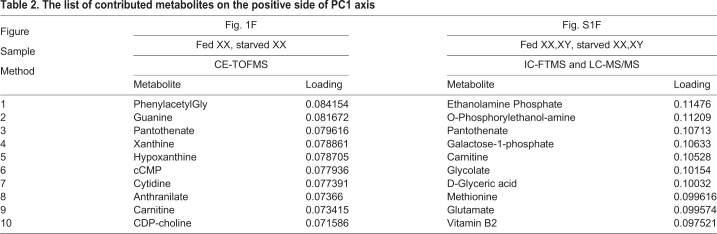
**The list of contributed metabolites on the positive side of PC1 axis**

To determine whether the increase in pantothenate contributes to sex reversal, larvae were treated with different pantothenate concentrations (20 µM, 200 µM and 2 mM) from 0–5 dph, under normal feeding conditions. However, sex reversal was not observed in adult XX medaka (Table 1). Contrary to the accumulation of pantothenate, downstream metabolites of pantothenate in this pathway did not show any increase under starvation conditions (Fig. 2B). This suggests that pantothenate accumulation is the result of low pantothenate pathway activity under starvation conditions, which may consequently lead to sex reversal (XX male).

To determine the involvement of the pantothenate pathway in sex reversal, we planned to inhibit this pathway. Pank is a rate-limiting enzyme in this pathway (Jackowski and Rock, 1981; Robishaw et al., 1982). We identified three putative *pank* genes (*pank1a*, *2* and *4*) in the medaka genome. *pank1b* and *pank3* are absent from the medaka genome in the corresponding syntenic region (Fig. S2A,B). Based on RT-qPCR analysis, *pank1a* transcript levels in the entire body were decreased by starvation (Fig. 2C). Whole-mount *in situ* hybridization (WISH) indicated that *pank1a* is mainly expressed in the liver (Fig. S2C,D) and *pank1a* expression was not detected in the gonads of either sex (Fig. S2E,F). These findings raise the possibility that suppressing the pantothenate pathway causes female-to-male sex reversal under starvation conditions.

To test this possibility, larvae were treated with a pantothenate kinase (Pank) inhibitor (50 µM) under normal feeding conditions from 0–5 dph. The amount of pantothenate increased in treated XX larvae (Fig. S3A). The pantothenate/4-phosphopantothenate ratio displayed a significant difference when compared with fed conditions (Fig. 2D). These data indicate that inhibitor treatment repressed pantothenate metabolism. The body length (Fig. S3B) and body weight (Fig. S3C) at 5 dph was not altered by inhibitor treatment, indicating that larvae grew normally under inhibitor treatment. The metabolic state under inhibitor treatment is more likely to resemble the starved state than the normally fed state (Fig. S3D,E). Furthermore, inhibitor treatment caused a decrease in lipids, especially TG, similar to that observed following starvation (Fig. 2E). Interestingly, the inhibitor-treated fish showed female-to-male sex reversal despite food consumption (Fig. 2F, Table 1). Together, these results indicate that pantothenate metabolism is involved in female-to-male sex reversal.

### Fatty acid synthesis contributes to female sex differentiation

**Fig. 3.**
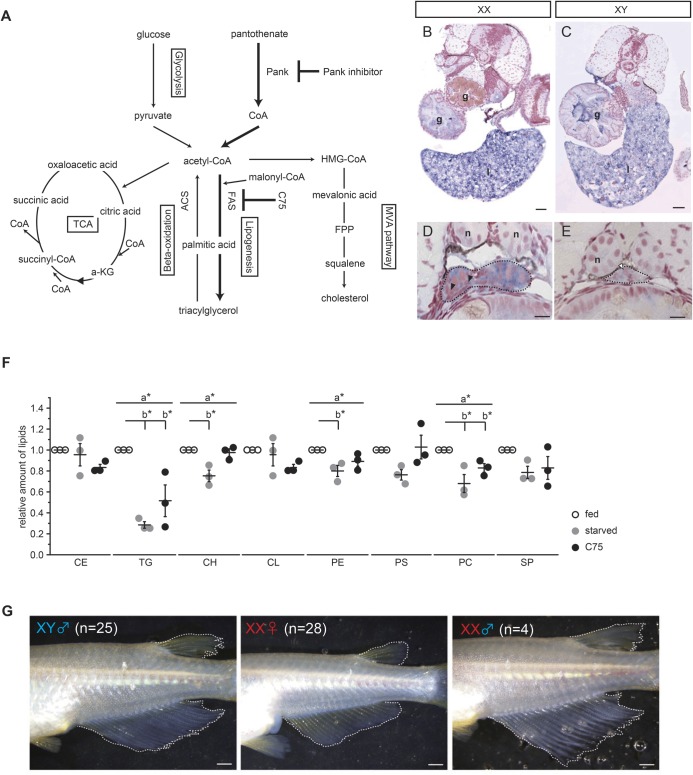
**Suppression of fatty acid synthesis contributes to female-to-male sex reversal.** (A) Metabolic pathways that utilize acetyl-CoA. (B–E) Analysis of *fasn* gene expression by whole-mount *in situ* hybridization at 5 dph. A strong signal is detected in the gut and liver of both sexes (B,C). XX gonads express *fasn* transcripts more than XY gonads. (D,E). Black dashed lines indicate the outline of the gonads. Black arrowheads indicate signal-positive germ cells. White arrowheads indicate signal-negative germ cells. g, gut; l, liver; n, nephric duct. Scale bars: 40 µm (B,C), 10 µm (D,E). (F) The relative amount of lipids in 5 dph whole larvae (*n*=3). The total lipids were extracted from four larvae. The amount of TG is decreased by C75 treatment, as seen in the starvation conditions. The values indicate the average and the bars indicate s.e.m. A one-way ANOVA followed by Dunnett’s test was used as statistical analysis. a*: *P*-value<0.05 in a two-way ANOVA. b*: *P*-value<0.05 in Dunnett’s test. (G) C75 treatment causes female-to-male sex reversal. Sex-reversed individual (XX ♂) indicates a male type of dorsal and anal fins. The genotyping result is shown in Table 1. Scale bars: 1 mm.

CoA, a final metabolite of the pantothenate pathway, is a critical substance that regulates other metabolic pathways, including the TCA cycle, the MVA pathway which is involved in cholesterol production and lipogenesis, among others (Leonardi et al., 2005) (Fig. 3A). Oil Red O staining showed that after 5 days of starvation, larvae are stained to a lesser degree than larvae that have been fed normally (Fig. 1C). HPTLC analysis indicated that several kinds of lipid were decreased by starvation and the inhibition of pantothenate metabolism (Figs 1D and 2E), which suggests that lipogenesis is involved in female-to-male sex reversal.

Most of the lipids, except cholesterol group, have a fatty acid chain, and fatty acid synthesis is the first step in lipogenesis. FAS, a multi-functional enzyme, produces all fatty acids ranging from butyric acid (4:0) to palmitic acid (16:0) (Liu et al., 2010). Only one *fasn* gene encoding FAS was found in the medaka genome (LG19). The other paralog that arose by teleost-specific whole genome duplication seems to have been lost from medaka (LG8) (Fig. S4A). The expression pattern of FAS gene (*fasn*) was determined by WISH. *fasn* was expressed in the gut and liver in both sexes of larvae at 5 dph (Fig. 3B,C). XX gonads showed strong signals of expression in somatic cells and all types of germlines (Fig. 3D). However, the signal was very weak in XY gonads (Fig. 3E).

Next, we examined the effect of an inhibitor of FAS (C75) (Rendina and Cheng, 2005). Larvae were treated with C75 (20 µg/ml) from 0–5 dph under normal feeding conditions. HPTLC indicated that the level of TG was decreased in larvae treated with C75 (Fig. 3F), suggesting that C75 inhibits FAS activity. C75 treatment did not cause any significant difference in body length (Fig. S4B). The metabolic state was again analyzed by PCA, following IC-FTMS (anionic metabolites) and LC-MS/MS (cationic metabolites). It showed that the clusters of C75 treatment samples were located near the clusters of fed samples (Fig. S4C,D), suggesting that the metabolic state under C75 treatment more closely resembles the normally fed state than the starved state. Surprisingly, female-to-male sex reversal was observed in a group subjected to C75 treatment despite a normal dietary intake that included lipids (Fig. 3G; Fig. S4E; Table 1). These data indicate that fatty acid synthesis is associated with to female-to-male sex reversal.

### *dmrt1* expression correlates with starvation and metabolic changes

**Fig. 4.**
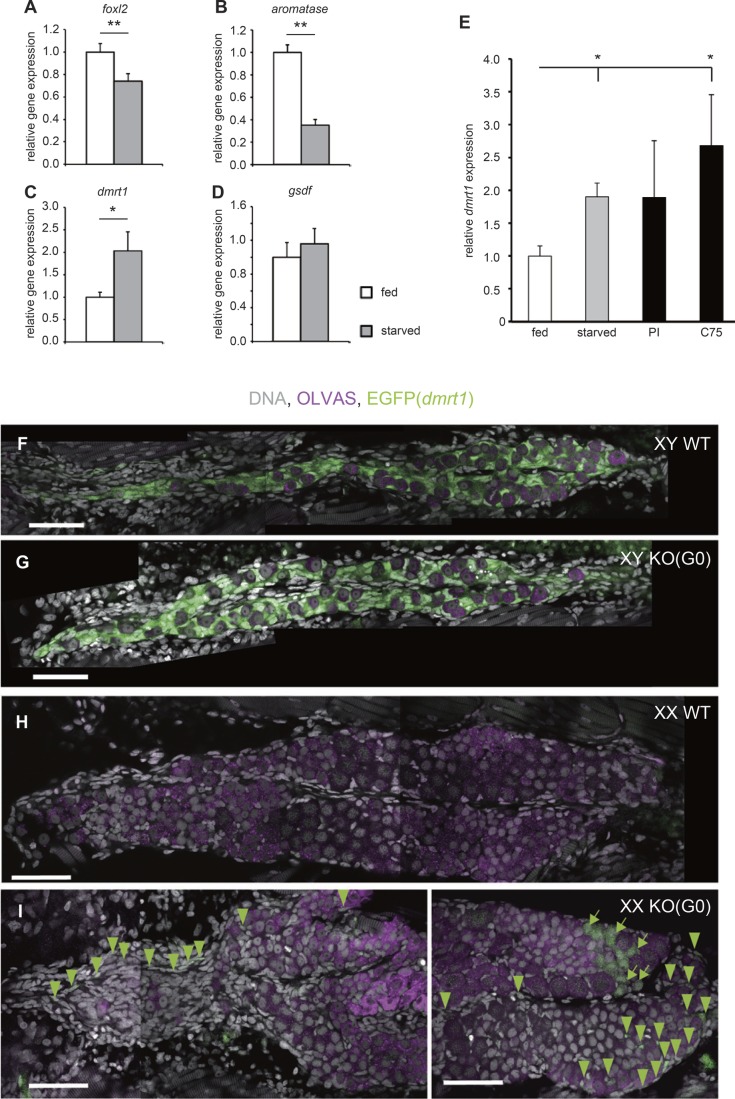
***dmrt1* is necessary for starvation-induced sex reversal.** (A–D) RT-qPCR analysis of *foxl2* (A), *aromatase* (B), *dmrt1* (C) and *gsdf* (D) transcripts extracted from one whole XX larva at 5 dph (fed *n=*16, starved *n=*13). Starvation causes the suppression of expression of female related-genes (*foxl2* and *aromatase*) and increase of expression of male related-gene (*dmrt1*). The values indicate the average and the bars indicate s.e.m. Student's *t*-test was used as statistical analysis. *P-value<0.05, **P-value<0.01. (E) RT-qPCR analysis of *dmrt1* transcripts extracted from one whole XX larva at 5 dph under fed (*n=*8), starved (*n=*6), 50 µM Pank inhibitor (*n=*7), and 20 µg/ml C75 (*n=*6) treatment conditions. *dmrt1* expression is increased by the treatment which induces female-to-male sex reversal. The values indicate the average and the bars indicate s.e.m. *P*-values from Student's *t*-test were corrected by Bonferroni correction. *P-value<0.05. (F–I) EGFP expression in gonad of *dmrt1* promoter-driven EGFP transgenic medaka at 5 dph. During normal development, EGFP expression is detected only in male gonads (F,G), but not in female gonads (H). EGFP positive cells are observed in *fasn* XX G_0_ larva at 5 dph (I). Scale bars: 50 µm. Green arrowheads indicate EGFP expression in somatic cells. Green arrows indicate EGFP expression in germ cells. The rate of gonad which had EGFP positive gonadal somatic cells; (F) wild type (WT) XY (*n*=7/7). (G) *fasn* G_0_ larva XY (*n*=7/7). (H) WT XX (*n*=0/15). (I) *fasn* G_0_ larva XX (*n*=3/5).

To determine whether starvation affects the expression of sex related-genes (female related-genes: *foxl2* and *aromatase*, male related genes: *gsdf* and *dmrt1*), the transcript level of each gene in the entire body was determined by RT-qPCR. Compared with larvae under normal feeding conditions, XX larvae after 5 days of starvation displayed decreased the expression of *foxl2* and *aromatase* (Fig. 4A,B). On the other hand, in male related-genes the expression of *dmrt1* was increased but not *gsdf* (Fig. 4C,D). The upregulation of *dmrt1* was also detected in larvae after 5 days of treatment with the Pank inhibitor and C75 (Fig. 4E). Fatty acid production is largely dependent on FAS activity. In mice, *fasn* knockout caused embryonic lethality at embryonic day 7.5 (E7.5) (Chirala et al., 2003). We planned to disrupt the *fasn* gene by using the CRISPR/Cas9 system and examine the G_0_ generation. Two different gRNAs were designed based on exon 4 of the *fasn* gene (Fig. S5A) and injected into one-cell- to two-cell-embryos derived from *dmrt1*-EGFP transgenic medaka (Herpin et al., 2010). EGFP signals in the transgenic line were detected in gonadal somatic cells surrounding germ cells in XY gonads (Fig. 4F,G), but EGFP was not observed in XX gonads (Fig. 4H). This demonstrates that EGFP in the transgenic line recapitulates *dmrt1* expression in male-supporting cells (Sertoli cells) (Herpin et al., 2010). The appearance of the *fasn* adult G_0_ medaka was very unique in that the mutant exhibited a short body length with a swollen belly (Fig. S5B). This appearance was observed in 78% (36/46) of *fasn* adult G_0_ medaka, suggesting that medaka raised from gRNAs injected eggs showed the specific phenotype at a high efficiency in G_0_ generation. We could not observe any female-to-male sex reversal among *fasn* adult G_0_ medaka (Table 1). Interestingly, however, EGFP signals were detected in gonadal somatic cells from three out of five *fasn* XX G_0_ mutants at 5 dph although the gonad showed a typical structure for XX gonads that develop ovaries with more mitotic germ cells than those in XY gonads (Fig. 4I).

To elucidate the involvement of *dmrt1* in female-to-male sex reversal under starvation conditions, larvae of *dmrt1*^−/−^ mutants (Δ13) (Fig. S6A–C) were subjected to 5 days of starvation after which the adult sex phenotype was examined at 6 months old. As we expected, the female-to-male sex reversal was not observed in starved *dmrt1* XX mutants (Table 1).

## DISCUSSION

**Fig. 5.**
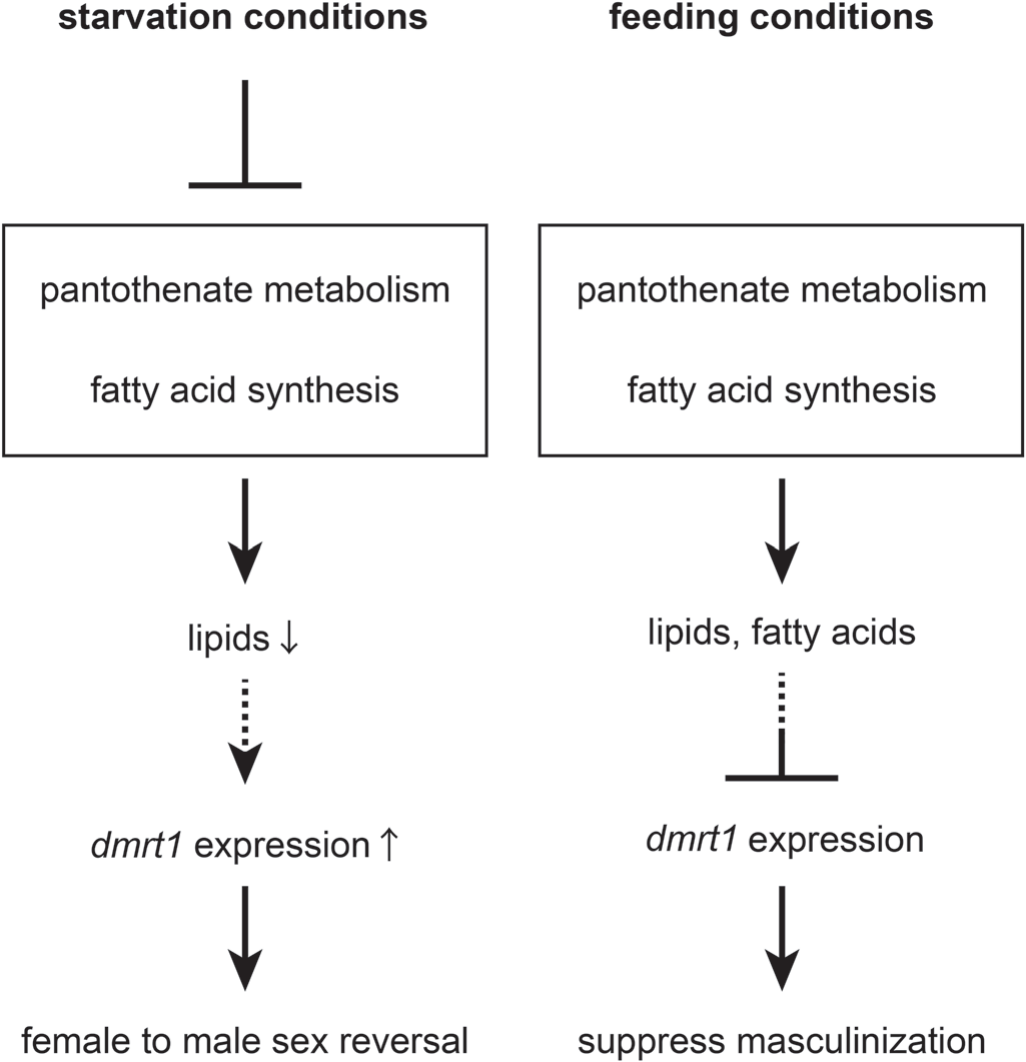
**Model mechanism underlying sex regulation under starvation and feeding conditions.** Under starvation conditions, fatty acid synthesis is downregulated through the suppression of pantothenate metabolism. Depletion of lipids causes ectopic *dmrt1* expression in XX somatic cells, which causes the genetic female (XX) to develop into a functional male. Conversely, under normal feeding conditions, XX larvae may suppress *dmrt1* expression through lipids.

In this study, we demonstrated that 5 days of starvation during sex differentiation causes female-to-male sex reversal through metabolic changes (Fig. 5). Starvation for 5 days suppressed the pantothenate pathway possibly via either *pank1a* downregulation, low PANK activity, or both. Although limited sample availability and the performance of our analytical apparatus did not allow detection of a difference in the amount of CoA, pharmacological experiments combined with metabolomic analyses suggest that starvation likely affects the amount of CoA, which is the final metabolite in the pantothenate pathway. Our HPTLC results also show that, in this situation, the level of lipids was decreased. Importantly, *fasn* G_0_ mutant XX larvae displayed the gonadal somatic cells expressing the *dmrt1*. This suggests that a portion of XX gonadal somatic cells was masculinized by *fasn* disruption. In turn, the inhibition of FAS activity leads to sex reversal to males. Collectively, these results support the idea that starvation turns genetic females into phenotypic males by lowering the rate of fatty acid synthesis. Finally, the male development gene, *dmrt1*, was ectopically expressed in female gonadal somatic cells following FAS inhibitor administration. Our findings suggest that, in wild-type medaka, fatty acids in genetic females to repress somatic masculinization by *dmrt1* expression. The regulation via lipids may represent a novel sex regulation system that responds to nutritional conditions.

In this context, it is interesting to note that *Daphnia*, a zooplanktonic crustacean, has an environmental sex determination system (Smith, 1915; Banta and Brown, 1929; Hobæk and Larsson, 1990; Kleiven et al., 1992). In *Daphnia pulex*, which propagates by parthenogenesis under long-day length conditions, the emergence of male with short-day length is associated with an increase in pantothenate levels. Contrary to our study, however, pantothenate administration induces masculinization (Toyota et al., 2015, 2016). The authors in Toyota et al. (2016), discussed that CoA, the last metabolite in the pantothenate pathway, was possibly utilized for methyl farnesoate production via the MVA pathway.

In addition to our results, medaka have been shown to undergo female-to-male sex reversal under high water temperature conditions during the period of fertilization to 5 dph (Sato et al., 2005; Hattori et al., 2007). HT conditions cause the elevation of cortisol and repression of *aromatase* expression (Hayashi et al., 2010; Kitano et al., 2012). *dmrt1* expression was induced in XX gonads by both HT and cortisol treatment (Adolfi et al., 2019). This may suggest some relation between cortisol and sex reversal. Because cholesterol can be a precursor of cortisol, it is interesting to have found the reduction in the amount of cholesterol in our starved samples. However, the detailed pathways that connect cortisol with sex reversal induced by lipid metabolism are still unknown.

Not all XX larvae showed sex reversal in our experiments (Table 1). We raised two possibilities to explain the low percentage of sex reversal. The first possibility is regarding the conditions applied for sex reversal. The 5 days of starvation may not be enough to cause sex reversal in all XX individuals. Under high-temperature conditions, the rate of sex reversal is increased depending on the temperature (Hattori et al., 2007; Adolfi et al., 2019). It suggests that a longer starvation period may increase the rate of sex reversal. However, in our case, over 5 days of starvation severely affects larval survival. The second possibility is that the competition between female and male mechanisms after 5 days of starvation influences the rate of sex reversal. The process of sex determination is generally understood as mutual repression between the two mechanisms, female and male determination (Capel, 2017). In this context, *dmrt1* has been regarded as a key gene for male determination. In fact, *dmrt1* expression is a prerequisite for sex reversal in this study. The induction of *dmrt1* by starvation, however, may be suppressed after normal feeding conditions are applied because the mechanism for feminization dominates that for masculinization in genetically female (XX) medaka. In other words, the rate of sex reversal would be a matter of maintenance of *dmrt1* expression in different sexes.

One important issue is to address groups of lipid metabolites associated with the change in sex. Polyunsaturated fatty acids are involved in gene expression via nuclear receptor families (Bordoni et al., 2006; Salter and Tarling, 2007). In addition, dietary short- and medium-chain saturated fatty acids are known as both signaling factors and energy sources (Schönfeld and Wojtczak, 2016; Kimura et al., 2019). Interestingly, 13% of FAS inhibitor (C75)-treated females showed sex reversal despite their dietary lipid intake (e.g. triacylglycerol, very long-chain fatty acid and cholesterol, etc.) under these conditions. This observation might suggest that *de novo* short- to long-chain saturated fatty acids (4:0–16:0) function in regulating *dmrt1* expression in the gonads of genetic females. Alternatively, the lowered lipid metabolite levels caused by a FAS inhibitor may trigger sex reversal.

Another important issue is identifying the tissue or organ that senses changes in the internal environment and produces key metabolites in response. WISH analysis for *pank1a* and *fasn* expression suggests that fatty acid synthesis, but not pantothenate metabolism, is activated in the XX gonad. CoA and/or its downstream metabolite are, however, produced in another organ, notably the liver, and may be conveyed to the gonad. Interestingly, genetically female (XX) gonads showed greater *fasn* expression than genetically male (XY) gonads. This may suggest that XX gonads produce more fatty acids to repress *dmrt1* expression (Fig. 5). Our results show that sex is regulated or maintained at a systemic level in response to environmental conditions.

## MATERIALS AND METHODS

### Medaka maintenance and treatment

OK-Cab, d-rR, and inbred strain fish (*Oryzias latipes*) were used for experiments. Fish were maintained on a 14 h light/10 h dark cycle at 25–28°C. Phenotypic sex was determined by observing the anal fin, dorsal fin and gonads under a stereomicroscope (Yamamoto, 1958; Nishimura et al., 2015, 2018). For starvation and pantothenate treatment, 50 larvae were treated with complete starvation or pantothenate (20, 200 and 2000 μM; 033-14165, Wako) in 1 l of water from 0 dph to 5 dph. For chemical (pantothenic kinase inhibitor (50 µM, 537983, Merck) and FAS inhibitor C75 (20 µg/ml, C5490, MilliporeSigma) treatment, 25–30 larvae were maintained in 30 ml water from 0–5 dph. The water with the compound was changed every day.

### TALEN-induced mutagenesis

The TALEN Targeter program (https://tale-nt.cac.cornell.edu/node/add/talen) was used to search the TALEN target site for *dmrt1.* TALEN assembly was performed as previously described (Nishimura et al., 2018). The left and right TALEN arms (250 ng/µl each) were injected into one- or two-cell-stage embryos from OK-Cab wild-type fish. G_0_ founders were crossed with wild-type adults. The mutant allele, Δ13, was identified in the F_1_ generation. Primer sets for genotyping the Δ13 allele are shown in Table S1.

### CRISPR/Cas9-induced mutagenesis

Cas9 mRNA was synthesized as previously described (Nishimura et al., 2018). Target sites in *fasn* were found using an online CRISPR/Cas9 target predictor (CCTop, http://crispr.cos.uni-heidelberg.de/). gRNA synthesis was performed following a modified version of the original protocol (Liang et al., 2015). Briefly, the template DNA for gRNA synthesis was amplified by PCR using six different primers. Cycling parameters were 95°C for 1 min; 32 cycles of 98°C for 5 s and 55°C for 15 s; 68°C for 1 min. All primers are described in Table S1. The resultant amplified fragment was used for *in vitro* transcription using the MEGAscript T7 kit (Thermo Fisher Scientific). gRNA was purified by ammonium acetate precipitation. The cocktail of *Cas9* mRNA (50 ng/µl) and two gRNAs (gRNA1, 100 ng/µl; gRNA2, 250 ng/µl) was injected into the one-cell- or two-cell-embryos derived from *dmrt1*-EGFP transgenic medaka (Herpin et al., 2010).

### *In situ* hybridization and immunohistochemistry

WISH and immunohistochemistry were performed as previously described (Nakamura et al., 2006). For Fig. 3B and C, the expression signals were observed using a BZ-X710 All-in-One Fluorescence Microscope (Keyence) with a 20X objective lens (CFI PlanApo Lambda 20X NA:0.75, Nikon). For Fig. 4F–I, the images of gonad were observed in three parts (anterior, middle and posterior) using a FV1000 confocal microscopy (Olympus) with 60X objective lens (UPLSAPO 60XO NA:1.35, Olympus). The three parts of gonadal images were manually tiled using a software of Illustrator (Adobe CS6) to generate an image of whole view on the gonad.

### RT-qPCR analysis

Total RNA was extracted from a whole 5 dph larva using TriPure Isolation Regent (Roche). cDNA was synthesized from 1 µg total RNA using ReverTra Ace qPCR RT Master Mix with gDNA Remover (Toyobo) and used as the resulting template for RT-qPCR. RT-qPCR was performed using the KOD SYBR qPCR Mix (Toyobo) and the StepOnePlus real-time PCR system (Thermo Fisher Scientific). Primer sets are described in Table S1.

### Metabolomic analysis

Tail tips from 5 dph medaka larva were dissected under basal saline solution (BSS, Kinoshita et al., 2009) and placed in lysis buffer for subsequent genotyping. The remainder of the body was frozen in liquid nitrogen as soon as possible. The samples were used for two different metabolome analyses: (1) CE-TOF-MS (anionic and cationic metabolites), and (2) IC-FTMS (anionic metabolites) and LC-MS/MS (cationic metabolites).

#### CE-TOF-MS

Sample preparation for CE-TOF-MS: the water-soluble metabolites derived from 20 medaka larvae at 5 dph were extracted in 500 μl of methanol containing 8 μM of two reference compounds (methionine sulfone for cation analysis and camphor 10-sulfonic acid for anion analysis) using a Retsch mixer mill MM310 at 27 Hz frequency for 1 min. The extracts were centrifuged at 15,000 ***g*** for 3 min at 4°C. The supernatant was transferred into a tube, to which 500 μl of chloroform and 200 μl of water were added to perform the extraction. The upper layer was evaporated for 30 min at 45°C using a centrifugal concentrator to obtain two layers. For removing high molecular weight compounds such as oligosaccharides, the upper layer was centrifugally filtered through a PALL Nanosep 3-kDa cutoff filter at 9100 ***g*** for 90 min at 4°C. The filtrate was dried for 120 min using a centrifugal concentrator. The residue (about 25 mg of each sample) was dissolved in 20 μl of water containing 200 μM internal standards (3-aminopyrrolidine for cation analysis and trimesic acid for anion analysis) that were used to compensate for migration time in the peak annotation step.

CE-TOF-MS conditions: all CE-TOF-MS experiments were performed using an Agilent G7100A CE Instrument (Agilent Technologies), an Agilent G6224A TOF LC/MS system, an Agilent 1200 Infinity series G1311C Quad Pump VL, the G1603A Agilent CE-MS adapter, and G1607A Agilent CE-ESI-MS sprayer kit. The software G1601BA 3D-CE ChemStation for CE was used on a G3335-64002 MH Workstation. Separations were carried out using a fused silica capillary (50 μm i.d.×100 cm total length) filled with either 1 M formic acid for cation analysis or 20 mM ammonium formate (pH 10.0) for anion analysis as the electrolyte. The capillary temperature was maintained at 20°C. The sample solutions were injected at 50 mbar for 15 s (15 nl). The sample tray was cooled below 10°C. Prior to each run, the capillary was flushed with electrolyte for 5 min. The voltage applied for separation was 30 kV. Fifty percent (v/v) methanol/water containing 0.5 μM reserpine was delivered as the sheath liquid at 10 μl/min. Electrospray ionization (ESI)-TOF-MS was conducted in positive ion mode for cation analysis or in negative ion mode for anion analysis; capillary voltage was set at 30 kV. A flow rate of heated dry nitrogen gas (heater temperature 300°C) was maintained at 10 l/min. The fragmentor, skimmer and Oct RFV voltage were automatically set to optimum values. Automatic recalibration of each acquired spectrum was performed using reference masses of reference standards. The methanol dimer ion ([2M+H]^+^, *m/z* 65.0597) and reserpine ([M+H]^+^, *m/z* 609.2806) for cation analysis or the formic acid dimer ion ([2M-H]^−^, *m/z* 91.0037) and reserpine ([M-H]^−^, *m/z* 607.2661) for anion analysis provided the lock mass for exact mass measurements. Exact mass data were acquired at a rate of 1.5 cycles/s over a 50–1000 *m/z* range. In every single sequence analysis (maximum 36 samples) on our CE-TOF-MS system, we analyzed the standard compound mixture at the start and the end of sample analysis. The detected peak area of the standard compound mixture was checked in point of reproducible sensitivity. Standard compound mixture was composed of major detectable metabolites, including amino acids and organic acids; this mixture was prepared anew at least once every 6 months. In all analyses in this study, there were no differences in the sensitivity of the standard compound mixture.

Data processing for CE-TOF-MS data: An original data file (.d) was converted to a unique binary file (.kiff) using the in-house software (nondisclosure). Peak picking and alignment of samples were performed automatically using another in-house software (nondisclosure). In contrast to the detected m/z and migration time values of standard compounds including internal standards, peaks were annotated automatically using the same software. For normalization, the individual area of the detected peaks was divided by the peak area of the internal reference standards.

#### IC-FTMS and LC-MS/MS

Sample preparation for IC-FTMS and LC-MS/MS analyses: metabolite extraction from a single larva (5 dph) for metabolomic analysis was performed as described previously (Oka et al., 2017; Miyazawa et al., 2017). Briefly, a single frozen larva together with internal standard compounds (see below) was homogenized in ice-cold methanol (500 μl) using a manual homogenizer (Finger Masher (AM79330), Sarstedt), followed by the addition of an equal volume of chloroform and 0.4 volume of ultrapure water (LC/MS grade, Wako). The suspension was centrifuged at 15,000 ***g*** for 15 min at 4°C. After centrifugation, the aqueous phase was ultrafiltered using an ultrafiltration tube (Ultrafree MC-PLHCC, Human Metabolome Technologies). The filtrate was concentrated using a vacuum concentrator (SpeedVac, Thermo Fisher Scientific). The concentrated filtrate was dissolved in 50 μl ultrapure water and used for IC-FTMS and LC-MS/MS analyses.

Quantification of metabolites by internal standards for IC-FTMS and LC-MS/MS analyses: internal standard compounds were used for concentration calculations. Internal standards were added to the tissue before extraction. 2-morpholinoethanesulfonic acid (MES) and 1,3,5-benzenetricarboxylic acid (trimesate) as internal standards for anionic metabolites are not present in tissues; thus, they serve as ideal standards. Loss of endogenous metabolites during sample preparation was corrected by calculating the recovery rate (%) for each sample measurement.

IC-FTMS for anionic metabolites: for metabolomic analysis focused on glucose metabolic central pathways, namely glycolysis, the TCA-cycle, and PPP, those anionic metabolites were measured using an orbitrap-type MS (Q-Exactive focus, Thermo Fisher Scientific), connected to a high performance ion-chromatography system (ICS-5000+, Thermo Fisher Scientific) that enabled us to perform highly selective and sensitive metabolite quantification owing to the IC-separation and Fourier Transfer MS principle (Hu et al., 2015). The IC was equipped with an anion electrolytic suppressor (Thermo Scientific Dionex AERS 500) to convert the potassium hydroxide gradient into pure water before the sample enters the mass spectrometer. The separation was performed using a Thermo Scientific Dionex IonPac AS11-HC, 4 μm particle size column. IC flow rate was 0.25 ml/min supplemented post-column with 0.18 ml/min makeup flow of MeOH. The potassium hydroxide gradient conditions for IC separation were as follows: from 1 mM to 100 mM (0–40 min), 100 mM (40–50 min), and 1 mM (50.1–60 min), at a column temperature of 30°C. The Q Exactive focus mass spectrometer was operated under an ESI negative mode for all detections. Full mass scan (*m/z* 70−900) was used at a resolution of 70,000. The automatic gain control target was set at 3×10^6^ ions, and maximum ion injection time was 100 ms. Source ionization parameters were optimized with 3 kV spray voltage and other parameters as follows: transfer temperature at 320°C, S-Lens level at 50, heater temperature at 300°C, Sheath gas at 36, and Aux gas at 10.

LC-MS/MS for cationic metabolites: the amount of cationic metabolites (amino acids) in a single larva (5 dph) was quantified using liquid chromatography-tandem mass spectrometry (LC-MS/MS). Briefly, a triple-quadrupole mass spectrometer equipped with an ESI ion source (LCMS-8040, Shimadzu Corporation) was used in the positive and negative-ESI and multiple reaction monitoring (MRM) modes. The samples were resolved on the Discovery HS F5-3 column (2.1 mmI.D.×150 mml, 3 μm particle, Sigma-Aldrich), using a step gradient with mobile phase A (0.1% formate) and mobile phase B (0.1% acetonitrile) at ratios of 100:0 (0–5 min), 75:25 (5–11 min), 65:35 (11–15 min), 5:95 (15–20 min), and 100:0 (20–25 min), at a flow rate of 0.25 ml/min and a column temperature of 40°C. MRM conditions for each amino acid were previously described (Oka et al., 2017).

### Oil Red O staining

The larvae were fixed with 10% formaldehyde for at least 10 h at 4°C. To prepare the stock solution, 60 mg of Oil Red O (Sigma-Aldrich, O0625-25G) dissolved in 20 ml 2-propanol (final concentration: 3 mg/ml, 12.3×10^2^ mol/l) and the stock was kept in the dark at room temperature (RT). As a staining solution, the stock was mixed with sterilized water (stock:sterilized water=3:2). To remove crystal, the solution was passed through a 0.22 nm filter (Merck Millipore). Sample was stained at RT for 2 h with shaking. After staining, the sample was washed three times with PBS at RT for 10 min three times, 60% 2-propanol and sterilized water at RT for 10 min each time.

### HPTLC analysis

Total lipid was extracted by the water:chloroform:MeOH (0.8:1:2) method (Bligh and Dyer, 1959; Mita and Ueta, 1989). The extract was loaded on HPTLC plate (Merck Millipore) and expanded by two different expanding solutions: (1) chloroform:methanol:acetic acid:formic acid:water=35:15:6:2:0.5, and (2) n-hexane:diethyl ether:acetic acid=70:30:1. For staining the lipids, a 3% copper acetate/phosphate solution was used (Macala et al., 1983; Mita et al., 1988). The image of the HPTLC plate was immediately taken by Printgraph (ATTO). The intensity of each lipid band was calculated using the software Fiji. Cholesteryl oleate, triolein, oleic acid, cholesterol, cardiolipin, L-a-phosphatidylethanolamine, L-a-phosphatidylserine, L-a-phosphatidylcholine, and sphingomyelin were used as standard lipids.

### Statistical analysis

The statistical significance of differences in the ratio of sex reversal was determined by Pearson's chi-squared test. A one-way ANOVA followed by Dunnett’s test was used for Figs 2E and 3F. For Figs 1D and 2D, Figs S3A, and B, a two-way ANOVA followed by Dunnett’s test was performed. Dunnett’s test is a comparison method between one control and other groups. As a control group, fed XX (Figs 1D and 2D), XX DMSO (Fig. S3A; Fig. 3B), and fed (Figs 2E and 3F) were used. *P*-value in Fig. 4E from Student's *t*-test was corrected by Bonferroni correction. Bonferroni correction multiplies the number of *t*-test trials to the *P*-value and compares it to the significance level (*P*<0.05). In Fig. 4E, we performed a comparison between fed and the other treatment conditions. Therefore, each *P*-value was corrected by multiplying by 3. The other tests of statistical significance we performed were Student's *t*-test. The all Student's *t*-test was performed by two-tails on Excel. R software was used for the other statistical analysis. Metabolomic data was analyzed by MetaboAnalyst version .4.0 (https://www.metaboanalyst.ca/). Metabolome (peak area) data was normalized by Auto scaling.

## Supplementary Material

Supplementary information
