# Supplementary material for: Starvation causes female-to-male sex reversal through lipid metabolism in the teleost fish, medaka (Olyzias latipes)
Source: Biol Open. 2020 Apr 3;9(4):bio050054. doi: 10.1242/bio.050054 (PMC7132775; doi:10.1242/bio.050054)
Supplement: Supplementary information [file biolopen-9-050054-s1.pdf]

Supplementary Figures

Figure S1

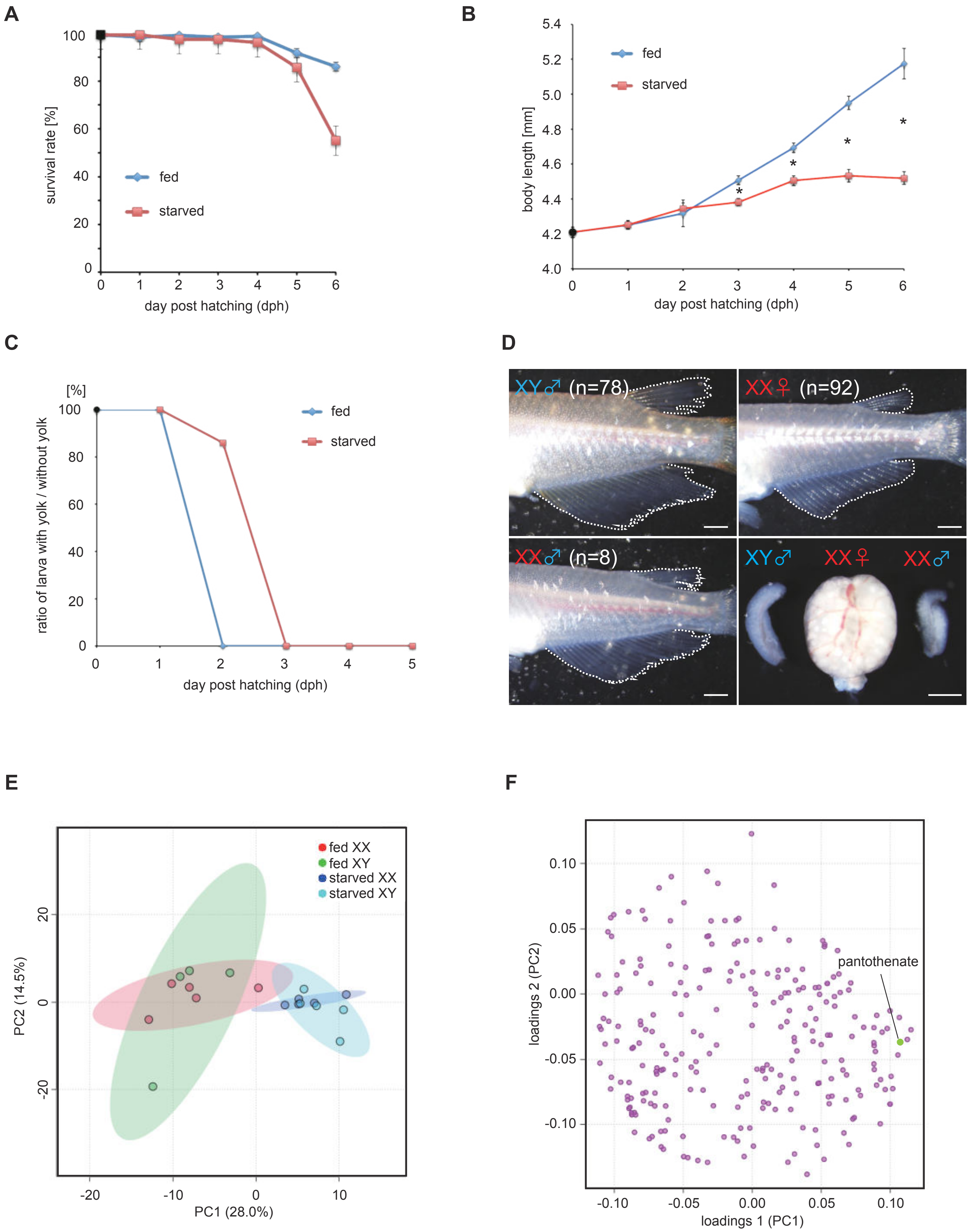

Figure S1. Starvation effects on medaka larvae.

(A) The survival rate during starvation treatment from 0-6 dph (0 dph *n* = 145, 1 dph fed *n* = 130, 1 dph starved *n* = 143, 2 dph fed *n* = 141, 2 dph starved *n* = 128, 3 dph fed *n* = 144, 3 dph starved *n* = 134, 4 dph fed *n* = 147, 4 dph starved *n* = 137, 5 dph fed *n* = 135, 5 dph starved *n* = 130, 6 dph fed *n* = 144, 6 dph starved *n* = 144). dph, day post hatching. The values indicate the average and the bars indicate s.e.m..

(B) Body length during starvation treatment from 0–6 dph. A significant difference appears at 3 dph (0 dph *n* = 45, 1 dph fed *n* = 30, 1 dph starved *n* = 43, 2 dph fed *n* = 41, 2 dph starved *n* = 28, 3 dph fed *n* = 44, 3 dph starved *n* = 34, 4 dph fed *n* = 47, 4 dph starved *n* = 37, 5 dph fed *n* = 35, 5 dph starved *n* = 30). The values indicate the average and the bars indicate s.e.m.. Student's t-test was used as statistical analysis. *P-value < 0.05.

(C) The presence of the yolk ball during starvation treatment from 0-5 dph. The yolk ball disappears until 2 dph under normal feeding conditions (0 dph *n* = 148, 1 dph fed *n* = 150, 1 dph starved *n* = 150, 2 dph fed *n* = 151, 2 dph starved *n* = 145, 3 dph fed *n* = 151, 3 dph starved *n* = 149, 4 dph fed *n* = 151, 4 dph starved *n* = 147, 5 dph fed *n* = 146, 5 dph starved *n* = 150). The values indicate the average.

(D) The appearance of dorsal and anal fins and gonads in the medaka d-rR strain. Sex-reversed individual indicates a male type of dorsal and anal fins with normal testis. The genotyping result is shown in Table 1. Scale bars: 1 mm.

(E) Principal component analysis based on metabolomic analysis (IC-FTMS and LC-MS/MS, 250 metabolites). Both XX and XY show different metabolic profiles depending on feeding conditions (fed XX *n* = 5, fed XY *n* = 4, starved XX *n* = 5, starved XY *n* = 5). Principal component, PC

(F) Loading plot of principal component analysis. A point indicates metabolite. Pantothenate shows a high contribution to the positive side of principal component 1. Principal component, PC

Biology Open • Supplementary information

Figure S2

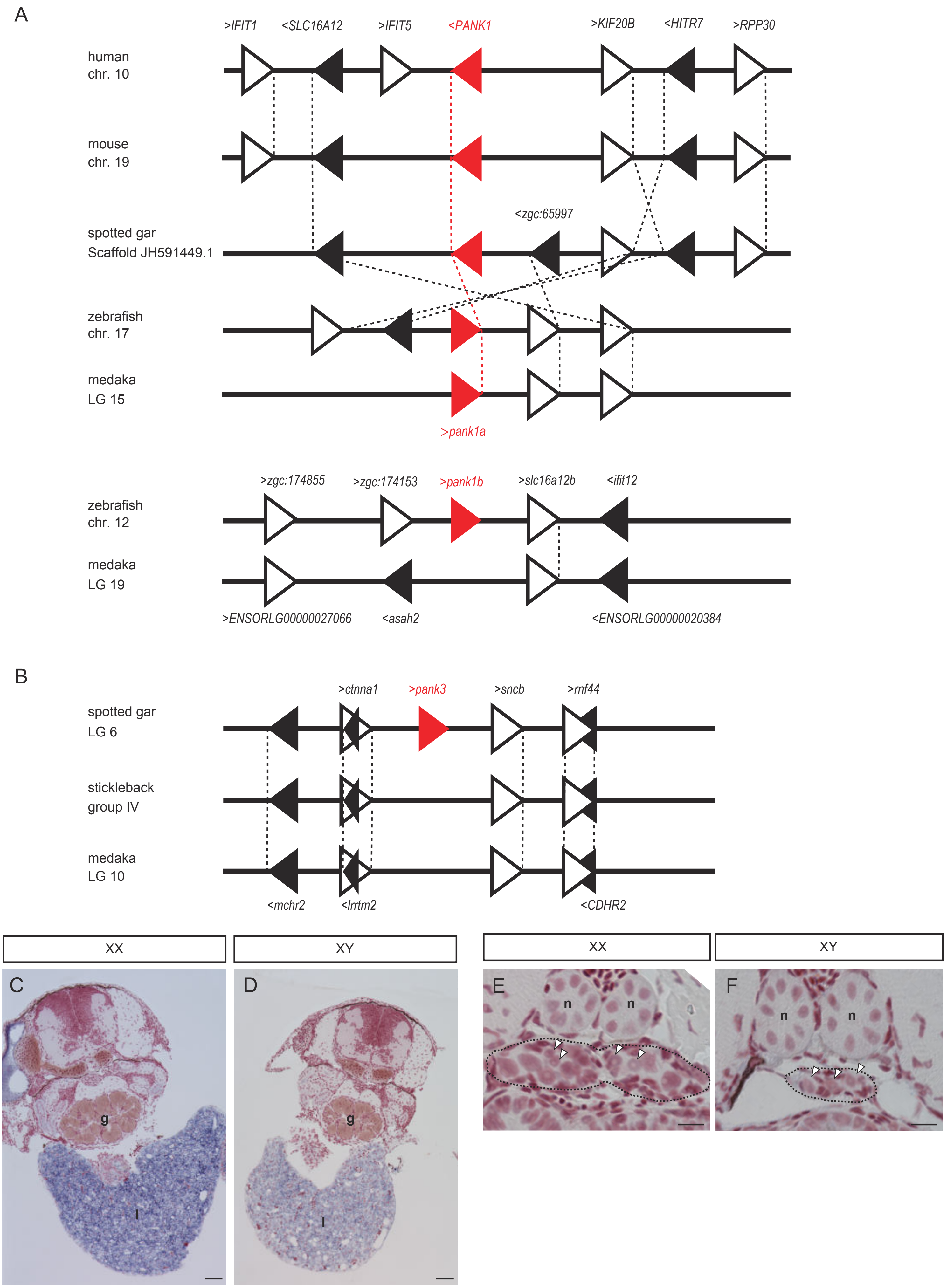

Biology Open • Supplementary information

Figure S2. Analysis of medaka *pank* genes.

(A) Synteny analysis for *pank1* gene. *pank1b* is not found in the syntenic region in the medaka LG19.

(B) Synteny analysis for *pank3* gene. *pank3* is not found in the syntenic region in the medaka LG10.

(C-F) *pank1a* expression was examined by whole mount *in situ* hybridization at 5 dph. Strong signals are detected in the gut and liver of both sexes (C, D). *pank1a* transcripts are not detected in the gonad (E, F). Black dashed lines indicate the outlines of the gonads. White arrowheads indicate germ cells with negative signals. g, gut; l, liver; n, nephric duct. Scale bars: 40 µm (C, D), 10 µm (E, F).

Biology Open • Supplementary information

Figure S3

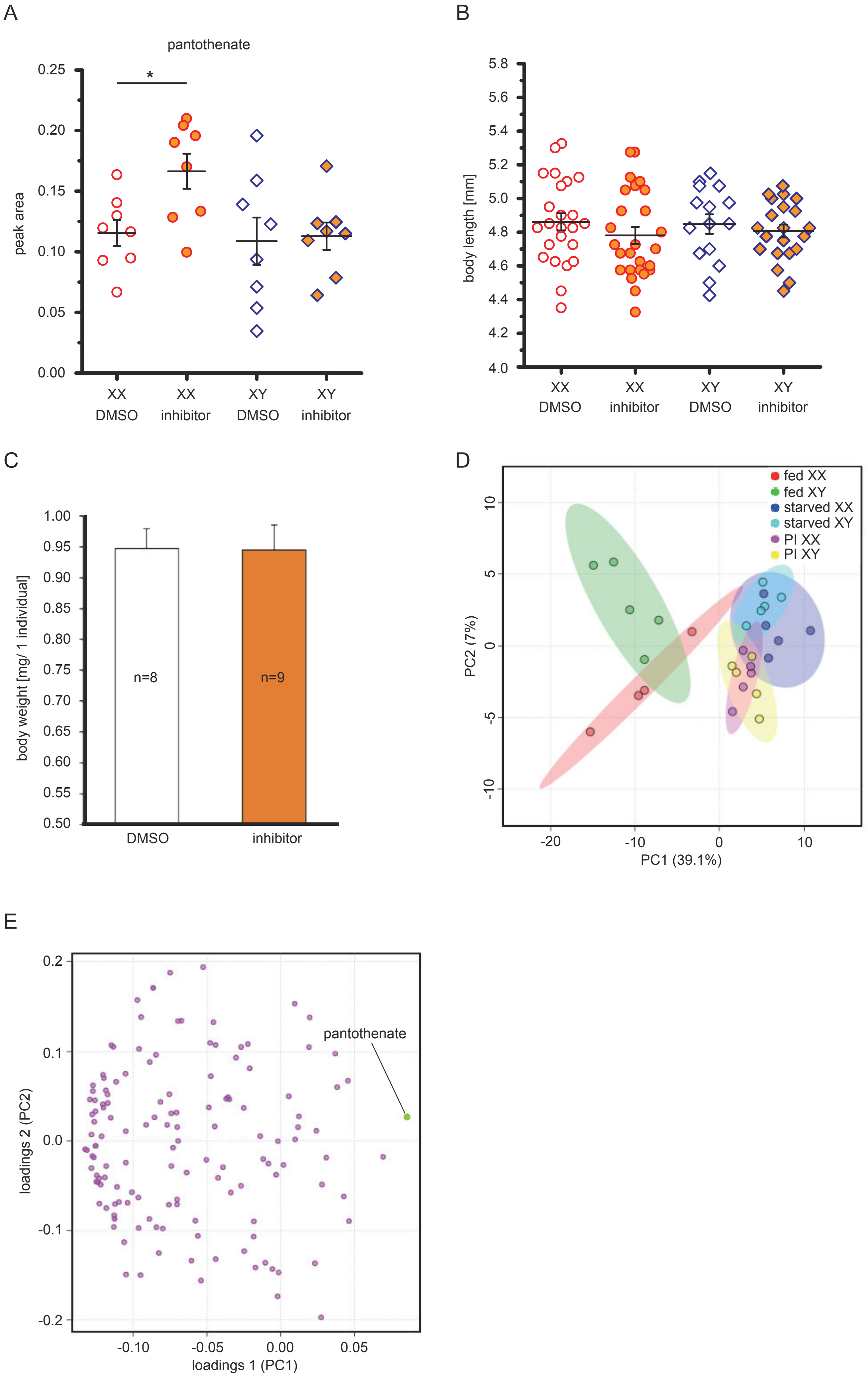

Biology Open • Supplementary information

Figure S3. Pank inhibitor treatment does not affect larval growth but causes pantothenate accumulation.

(A) The amount of pantothenate after 5 days of Pank inhibitor treatment. XX larvae treated with Pank inhibitor indicate the increase of pantothenate (XX DMSO n = 8, XX inhibitor n = 8, XY DMSO n = 8, XY inhibitor n = 8). The vertical axis indicates the peak area of pantothenate in the spectrogram of IC-FTMS analysis. The values indicate the average and the bars indicate s.e.m.. A two-way ANOVA followed by Dunnett test was used as statistical analysis. *: *P-value* < 0.05 in Dunnett test.

(B) Body length at 5 dph under Pank inhibitor (50 µM) treatment. Treated larvae show normal body length (XX DMSO n = 24, XX inhibitor n = 26, XY DMSO n = 15, XY inhibitor n = 21). The values indicate the average and the bars indicate s.e.m.. A two-way ANOVA followed by Dunnett test was used as statistical analysis.

(C) Body weight after 5 days of Pank inhibitor treatment. The weight of a single larva was calculated from the results of measurement using 4-6 larvae. (DMSO = 8 times, inhibitor = 9 times). Larvae treated with Pank inhibitor show normal body weight. The values indicate the average and the bars indicate s.e.m.. Student's t-test was used as statistical analysis.

(D) Principal component analysis based on metabolomic analysis (IC-FTMS, 141 metabolites). Pank inhibitor (PI, 50 µM)-treated larvae show a starved-like metabolic profile (fed XX n = 4, fed XY n = 5, starved XX n = 5, starved XY n = 5, PI XX n = 5, PI XY n = 5). One dot indicates a data set of a single larva.

(E) Loading plot of principal component analysis. A point indicates metabolite. Pantothenate shows a high contribution to the positive side of principal component 1. Principal component, PC

Biology Open • Supplementary information

Figure S4

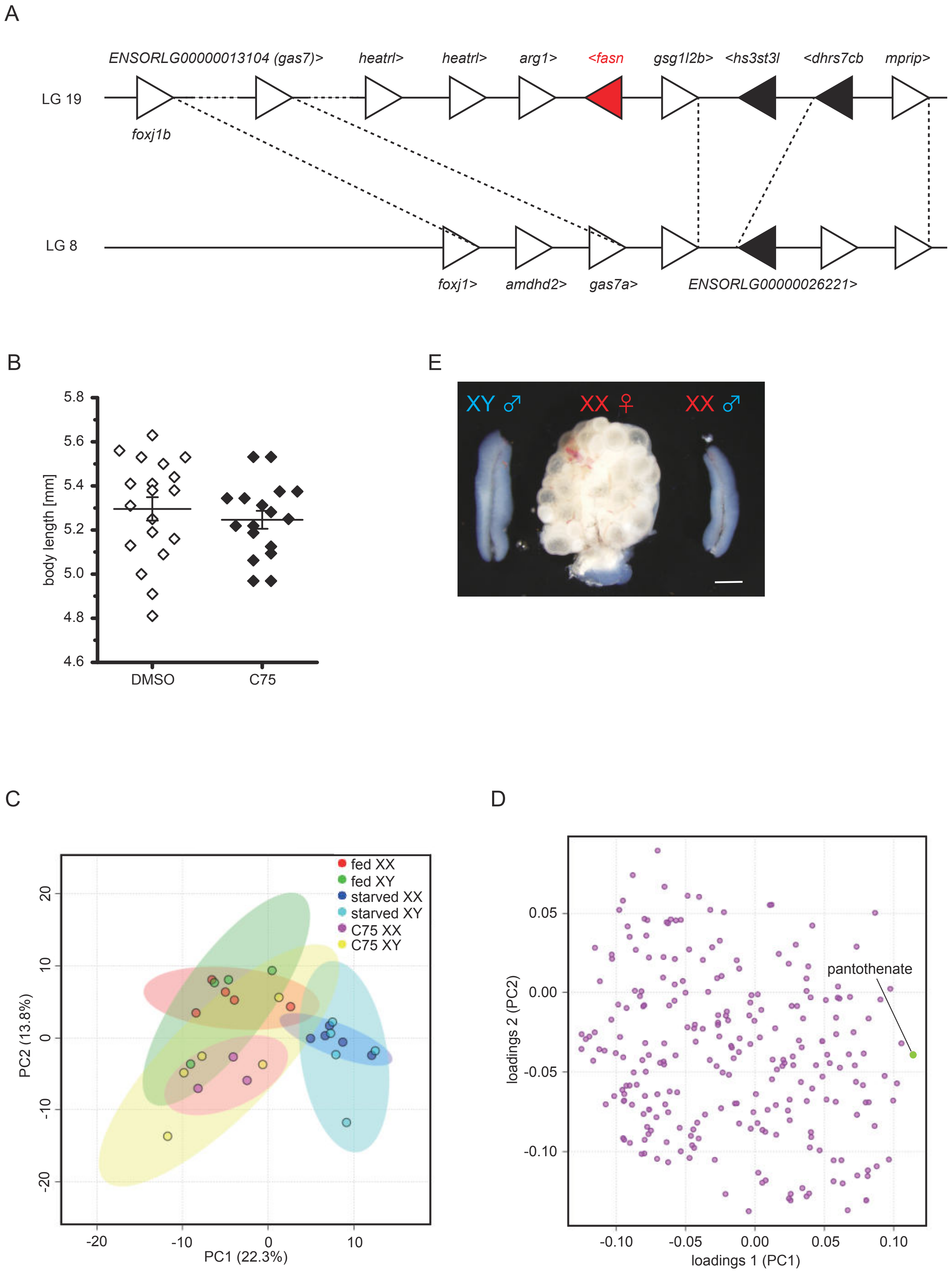

Biology Open • Supplementary information

Figure S4. The effects of C75 (FAS inhibitor) treatment.

(A) Synteny analysis for *fasn* gene on medaka genome. The paralogue for *fasn* is not found on the medaka LG8.

(B) Body length at 5 dph under C75 treatment (20 µg/mL). Treated larvae show normal body length (DMSO *n* = 19, C75 *n* = 17). The values indicate the average and the bars indicate s.e.m.. Student's t-test was used as statistical analysis.

(C) Principal component analysis based on metabolic analysis (ICFTMS, LC-MS/MS, 250 metabolites). C75 (20 µg/mL)-treated larvae do not show a starved-like metabolic profile (fed XX *n* = 5, fed XY *n* = 4, starved XX *n* = 5, starved XY *n* = 5, C75 XX *n* = 3, C75 XY *n* = 5).

(D) Loading plot of principal component analysis. A point indicates metabolite. Pantothenate shows a high contribution to the positive side of principal component 1. Principal component, PC

(E) Gonad from C75-treated fish (most right). Sex-reversed individuals have normal testis. Scale bars: 1 mm.

Biology Open • Supplementary information

Figure S5

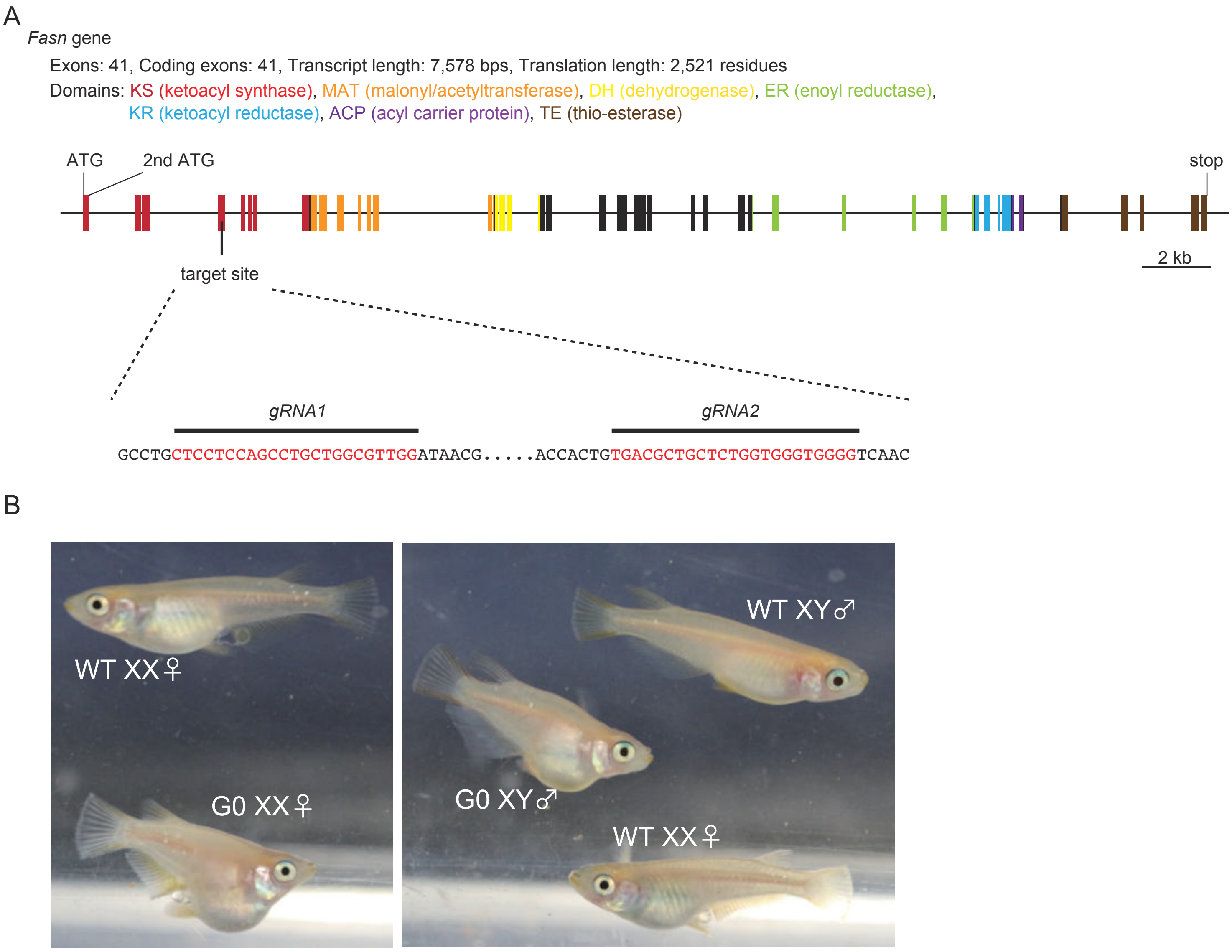

Figure S5. fasn disruption by CRISPR/Cas9 system.

(A) Design of gRNAs for disruption of fasn gene. The two different gRNAs were designed based on exon 4 of the fasn gene.

(B) The body appearance of G0 adult medaka. The G0 adult medaka show a short body length with a swollen belly.

Biology Open • Supplementary information

Biology Open (2020): doi:10.1242/bio.050054: Supplementary information

Figure S6

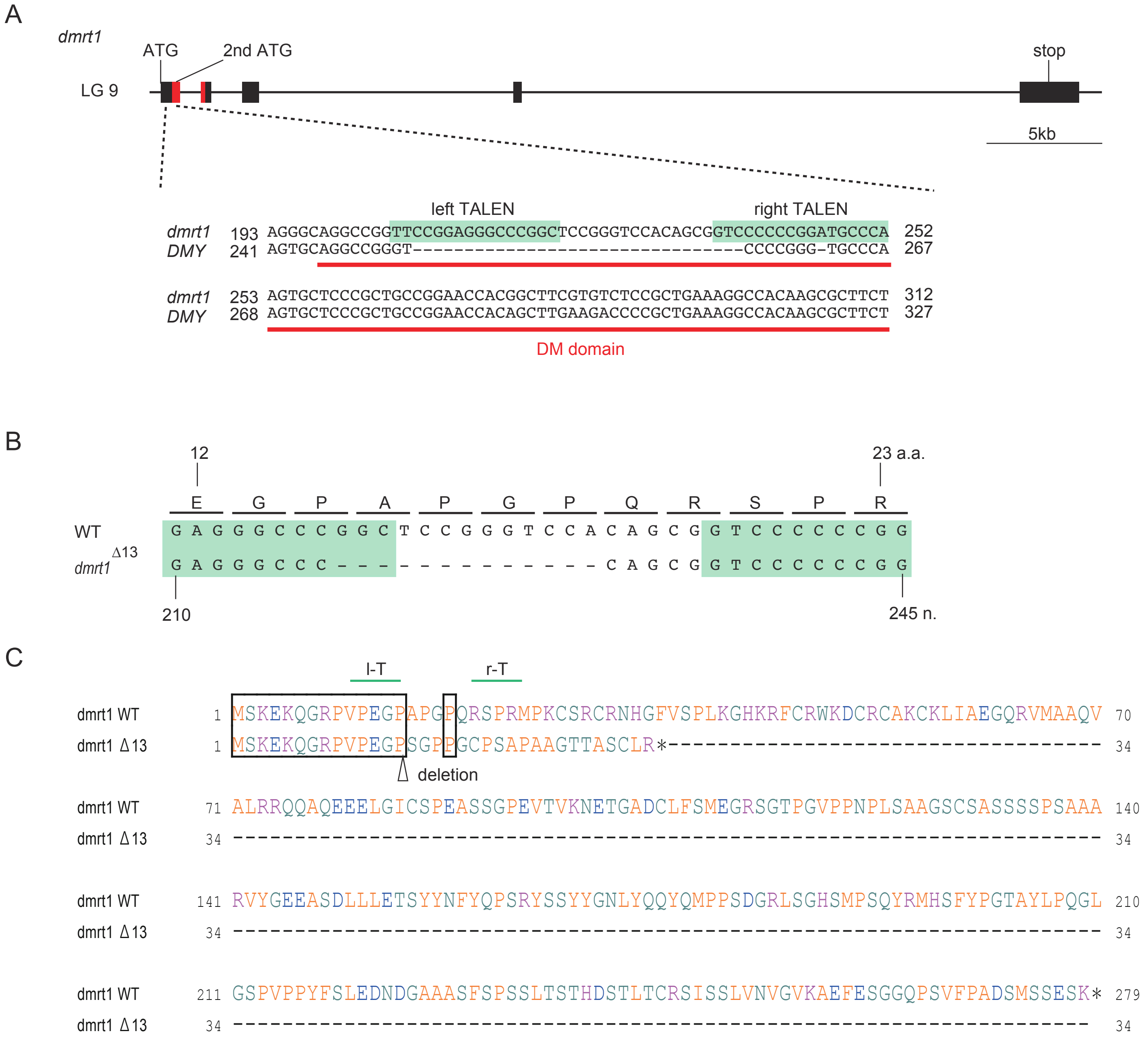

Figure S6. Design of TALEN for dmrt1 disruption.

(A) The location of left and right TALEN arms on the dmrt1 gene. TALEN was designed based on exon 1 of the dmrt1 gene.

(B) The sequence of dmrt1 in Δ13 line.

(C) The expected amino acid sequence of dmrt1 in Δ13 line. ＊: stop codon.

Biology Open • Supplementary information

Table S1. Primers and TaqMan probes used in this study.

Experiment	primer name	sequence (5'-3')
	tq-dmy-F	CGGTAAAATTGACGCACAGCAT
	tq-dmy-R	GGTGATTACTTGTGTTTCCACATTT
	dmy-FAM (TaqMan probe)	TGGCTTCACCGTTGGA
genotyping	tq-cyp19a-F	TGGCACAGCCAGCAACTATTA
	tq-cyp19a-R	TCCGTTGATCCACACTCGAA
	cyp19a-VIC (TaqMan probe)	ACAACAAATATGGAGACTT
	dmrt1-typing-F1	TCCTGTACAAGTGACCCCGC
	dmrt1-typing-R1	TTCAGCGGAGACACGAAGCC
	fasn probe F	TGGAGATCGGGAAGTACGAC
In situ	fasn probe R	GGCCAGTGAATTGTAATACGACTCACTATAGGGAACTGGTCCAACACCTCCAG
hybridization	pank1a probe F	CGTCCACTTGGAGCTGAGAA
	pank1a probe R	GGCCAGTGAATTGTAATACGACTCACTATAGGGTCACGAGTCATCTGTTGTC
	fasn gRNA1 F	TAATACGACTCACTATAGGCCTCCAGCCTGCTGGCGT
	fasn gRNA1 R	TTCTAGCTCTAAAACACGCCAGCAGGCTGGAGG
fasn KO	fasn gRNA2 F	TAATACGACTCACTATAGGACGCTGCTCTGGTGGGTG
	fasn gRNA2 R	TTCTAGCTCTAAAACCACCCACCAGAGCAGCGT
	80nt-crRNA-F	GTTTTAGAGCTAGAAATAGCAAGTTAAAATAAGGCTAGTCCGTTATCAACTTGAAAAAGTGGCACCGAGTCGGTGCTTTT
	80nt-crRNA-R	AAAAGCACCGACTCGGTGCCACTTTTTCAAGTTGATAACGGACTAGCCTTATTTTAACTTGCTATTTCTAGCTCTAAAAC
	T7-F	TAATACGACTCACTATAG
	crRNA-R	AAAAGCACCGACTCGGTG
	fasn check F	CCAGAGGAACGGTTGTTTTC
	fasn check R	TGAGCATGCCCAGTTTCAT
	b-actin F	TGGCGCTTGACTCAGGATTT
	b-actin R	GCAGATGCCTGGGGTGTTTA
	cyp19a1a F	CGCACAGAGTTCTTCCACAAAG
	cyp19a1a R	AACGGCTGGAAATAACGACGA
	foxl2 F	CACGCTGTCTGGCATCTACC
	foxl2 R	TCGTTGAGGCTCAGGTTGTG
	dmrt1 F	AGGGCCCGGCTCCGGCTCCA
RT-qPCR	dmrt1 R	TTCAGCGGAGACACGAAGCC
	gsdf F	TCCATGGCCACCGAGGTCTT
	gsdf R	CCGAGGAATTGCAGAGAGCAC
	pank1a F	GCTTTGGAAATGGCCAGTAA
	pank1a R	CATGTGACCAAAGCTGGATG
	pank2 F	CACCCTCTCCATGAAGCTGT
	pank2 R	GATGCAGCAACTCAAGCAAG
	pank4 F	TCACCGTCAGGAGTCTGTTG
	pank4 R	TCGTTCTCCCTCTGCTTGAT

Biology Open • Supplementary information
